# Evaluation of Cytotoxicity of Hyaluronic Acid/Chitosan/Bacterial Cellulose-Based Membrane

**DOI:** 10.3390/ma16145189

**Published:** 2023-07-24

**Authors:** Duangkamol Dechojarassri, Tomoki Okada, Hiroshi Tamura, Tetsuya Furuike

**Affiliations:** 1 Faculty of Chemistry, Materials and Bioengineering, Kansai University, Osaka 564-8680, Japan; 2 Organization for Research and Development of Innovative Science and Technology (ORDIST), Kansai University, 3-3-35 Yamate-cho, Suita, Osaka 564-8680, Japan

**Keywords:** layer by layer, alginate, wound dressing

## Abstract

Novel wound dressing materials are required to non-cytotoxic with a viable cell ratio of above 92%. Herein, the cytotoxicity of hyaluronic acid/chitosan/bacterial cellulose-based (BC(CS/HA)) membranes are evaluated and compared to that of alginate/chitosan/bacterial cellulose-based (BC(CS/Alg)) membranes was investigated. Multilayer membranes with up to ten CS/HA or CS/Alg layers were prepared using the layer-by-layer (LBL) method. Scanning electron microscopy showed that the diameters of the fibers in the BC(CS/Alg) and BC(CS/HA) membranes were larger than those in a BC membrane. The cytotoxicity was analyzed using BALB-3T3 clone A31 cells (mouse fibroblasts, 1 × 10^4^ cells/well). The BC(CS/HA)_5_ and BC(CS/HA)_10_ membranes exhibited high biocompatibility, with the cell viabilities of 94% and 87% at 5 d, respectively, compared to just 82% for the BC(CS/Alg)_5_ and BC(CS/Alg)_10_ membranes with same numbers of layers. These results suggested that BC(CS/HA)_5_ is a promising material for wound dressings.

## 1. Introduction

Nontoxicity is an essential property of biomaterials because they are in contact with living cells. Biomaterials should not exhibit toxicity or cause allergic reactions, and thus, natural materials such as chitosan (CS), bacterial cellulose (BC), alginate (Alg), and hyaluronic acid (HA) have received more attention than synthetic materials. CS is a linear polysaccharide consisting of β-(1-4)-linked d-glucosamine and *N*-acetyl-d-glucosamine units. It is obtained by the *N*-deacetylation of chitin and is the most common chitin derivative. CS-based biopolymers have excellent properties, such as biodegradability, biocompatibility, low cytotoxicity, and antimicrobial activity. Due to its unique properties, CS has attracted attention for biomedical applications [1,2,3,4,5]. The use of CS in wound healing has also been investigated [6,7,8]. BC and microbial cellulose also exhibit excellent biocompatibility, as well as good mechanical properties, high crystallinity, and film-formation abilities [9]. BC has been proven to help skin recover from burns and chronic wounds [10,11,12]. Alg, also called alginic acid, is a linear unbranched polysaccharide comprising (1–4)-linked β-d-mannuronic acid and its epimer α-d-guluronic acid. Owing to its outstanding properties (antimicrobial activity, gelation, biocompatibility, moisture adsorption, and hydrophilicity), Alg has been used in the food, cosmetic, pharmaceutical, and biomedical industries [13,14]. Alg-based materials are also promising for use as wound dressings [15,16,17,18].

For biomedical applications, cytotoxicity is a key factor for material design. In 2013, Archana et al. reported that the materials with cell viabilities of more than 75% could be applied as non-cytotoxic materials in various biomedical applications [19]. Later, in 2019, Xue et al. proposed a higher viable cell ratio criterion of 92% for wound dressing materials [20]. Therefore, during the development of novel wound dressing materials, a viable cell ratio of above 92% is required.

Layer-by-layer (LBL) technology has been successfully applied to prepare multilayer films. Driving forces, including electrostatic interactions, hydrogen bonds, Schiff base linkages, host–guest interactions, antibody–antigen interactions, and charge transfer interactions play essential roles in the LBL technique [21]. The amino and hydroxyl groups of CS can form bonds via electrostatic interactions with the negatively charged groups of other materials. For example, the –NH_3_^+^ group of CS interacts with the –COO^−^ group of Alg to form bilayer or multilayer films via the LBL technique [22,23,24]. In 2012, Gomes et al. successfully prepared an CS/Alg multilayer membrane on cotton gauze using the LBL method [25]. They later added antimicrobial peptides to the CS/Alg multilayer membrane and investigated its suitability for medical applications [26]. More recently, our group prepared an alginate/chitosan/bacterial cellulose-based (BC(CS/Alg)) membrane via the LBL method for electric capacitor applications [24]. Other wound healing materials have also been prepared by the LBL method [27,28]. Moreover, Mandapalli et al. prepared CS/Alg multilayer film by the LBL method and loaded it with the drug pirfenidone (PFD). Notably, the prepared film provided a higher wound healing efficiency than polyethylene glycol and CS hydrogels loaded with PFD [29]. This higher wound healing efficiency suggests that the multilayer materials by the LBL method are appropriate for producing novel wound dressing.

HA is a linear polysaccharide of disaccharide units composed of *N*-acetyl-d-glucosamine and d-glucuronic acid. HA has antimicrobial, antiadhesive, biodegradable, biocompatible, immunostimulatory, lubricating, and viscoelastic properties [30] and has been used in many biomedical fields, including bone generation, drug delivery, dental applications, and scaffolds for tissue engineering [31,32,33,34]. Li et al. reported that a BC/HA composite film with 0.1% HA showed better wound healing performance than both a pristine BC film and cotton gauze [35]. CS also interacts with HA via electrostatic interactions, making it possible to prepare CS/HA multilayer films by the LBL technique [21,36]. Therefore, in this study, we prepared and characterized hyaluronic acid/chitosan/bacterial cellulose-based (BC(CS/HA)) membranes and alginate/chitosan/bacterial cellulose-based (BC(CS/Alg)) membranes by the LBL method and compared their properties for wound dressing applications, including their cytotoxicity, morphology, tensile strength, functional groups, moisture content, and thermal stability.

## 2. Materials and Methods

### 2.1. Materials

Gluconacetobacter xylinus (G. xylinus; ATCC 53582) was purchased from Sumishin Pharmaceuticals International Corporation (Tokyo, Japan). CS FL-80 (lot. 181003, DAC87, 6 mPa·s) was provided by Koyo Chemical Co., Ltd. (Osaka, Japan). Peptone and yeast extract (extract of autolyzed yeast cells) were purchased from Becton, Dickinson and Company (Franklin Lakes, NJ, USA). Alg (sodium salt; 80–120 cP), HA (sodium salt), sodium hydrogen carbonate (NaHCO_3_), and l-glutamic acid were purchased from Fujifilm Wako Pure Chemical Corporation (Japan). Eagle’s minimum essential medium (EMEM) was purchased from Nissui Pharmaceutical Co., Ltd., Tokyo, Japan. Fetal bovine serum (FBS) was purchased from Biowest company, Miami, FL, USA. BALB-3T3 clone A31 cells (mouse fibroblasts, Resource No. RCB0005, Lot No. 16, Passage: 3) were purchased from Riken BRC Cell Bank, Ibaraki, Japan. All chemicals were used without further treatment.

### 2.2. Preparation of BC Membranes

Hestrin–Schramm (HS) medium containing 2% d-glucose, 0.5% yeast extract, 0.5% peptone, 0.115% sodium hydrogen phosphate, and 0.27% citric acid in deionized (DI) water (*w*/*v*) was sterilized at 121 °C for 15 min. *G. xylinus* (1 mL) was added to 200 mL of HS medium and incubated at 37 °C for 7 d. After that, 1 mL of pre-culture was added to 20 mL of fresh HS medium and poured into a plastic Petri dish with a diameter of 8.5 cm. The mixture was cultured at 37 °C for 7 d to produce circular membranes. Subsequently, the obtained BC membrane was washed with DI water and then treated with 4% NaOH solution at 97 °C for 3 h. The treated BC membrane was washed with DI water until the pH of the filtrate was 7 to obtain the circular BC membrane with a diameter of 8.5 cm.

### 2.3. Preparation of Chitosan-Modified Bacterial Cellulose (BC/CS) Membranes by Periodate Oxidation

During LBL membrane formation, the material to be coated should be positively or negatively charged. Therefore, in this study, the BC membrane surface was modified by coating with CS using periodate oxidation, according to the method of Kotatha et al. [24].

**Figure 1:**
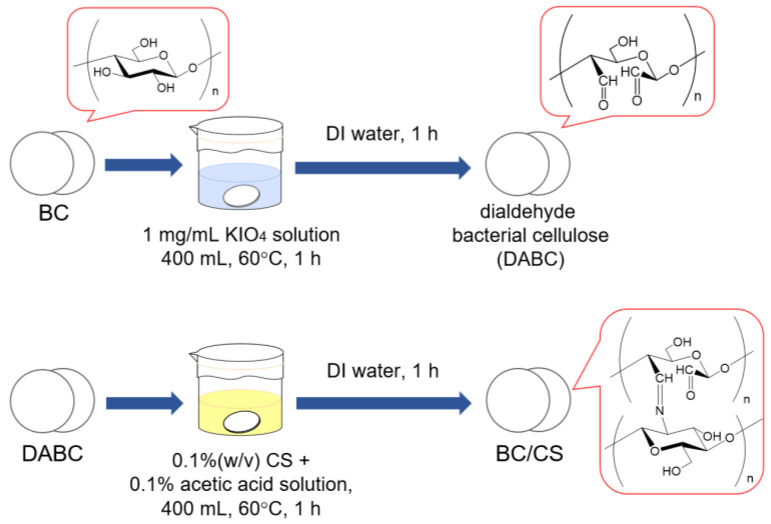
Preparation of the BC/CS membrane.

First, the swollen circular BC membranes prepared in Section 2.2 were immersed and stirred in 400 mL KIO_4_ aqueous solution (1 mg/mL) in a water bath at 60 °C for 1 h. The BC membrane was washed with DI water for 1 h and then soaked in DI water overnight. In this step, BC was converted to dialdehyde BC (DABC). Second, the CS powder was added to 400 mL of 0.1% acetic acid to obtain a CS concentration of 0.1% (*w*/*v*). Then, the DABC membrane was immersed in the 0.1% CS solution and stirred at 60 °C for 1 h, followed by washing with DI water for 1 h. Finally, the membrane was allowed to stand in DI water overnight. The preparation of the BC/CS membranes is depicted in Figure 1. 

### 2.4. Preparation of Chitosan–Alginate and Chitosan–Hyaluronic Acid Laminated BC Membranes Using the LBL Method

**Figure 2:**
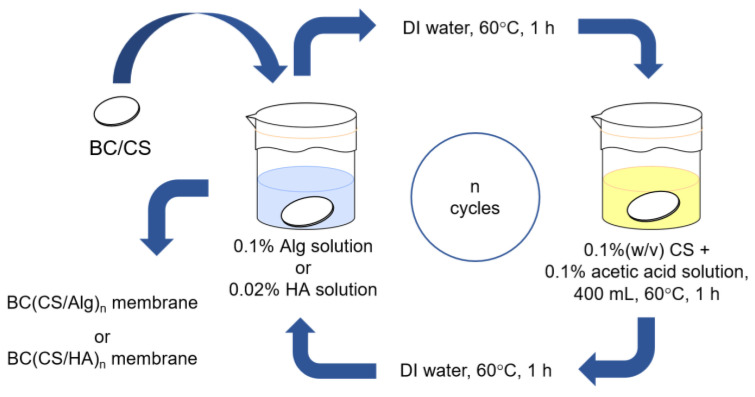
Preparation of the laminated membranes using the LBL method.

The preparation of the CS/Alg- and CS/HA-laminated BC membranes using the LBL method is shown in Figure 2. Five types of membranes were prepared, the details of which are listed in Table 1.

#### 2.4.1. Preparation of Chitosan-Alginate Laminated Membranes

Alg and CS were laminated onto the prepared BC/CS membranes using the LBL method. Alg powder (0.4 g) was added to 400 mL of DI water to prepare a 0.1% Alg solution. The BC/CS membrane prepared in Section 2.3 was immersed in the 0.1% Alg solution and stirred at 60 °C for 1 h. The membrane was then removed from the solution and washed with 400 mL of DI water at 60 °C for 1 h. The resulting membrane was named BS(CS/Alg)_1_. For the second-round coating, the BS(CS/Alg)_1_ membrane was immersed in a 0.1% CS solution and stirred at 60 °C for 1 h, followed by washing with DI water at 60 °C for 1 h. Subsequently, the membrane was immersed in the 0.1% Alg solution with stirring at 60 °C for 1 h and washed with 400 mL of DI water at 60 °C for 1 h. The membrane was laminated with up to 10 Alg/CS layers (BS(CS/Alg)*_n_*, where *n* is the number of coating cycles) by repeating the coating cycle up to 10 times. Finally, the circular BC(CS/Alg)_5_ and BC(CS/Alg)_10_ membranes with a diameter of 8.5 cm was obtained and the thickness of the membranes is shown in Table 2.

#### 2.4.2. Preparation of Chitosan-Hyaluronic Acid-Laminated Membranes

HA and CS were laminated onto the prepared BC/CS membranes using the LBL method. HA powder (0.08 g) was added to 400 mL of DI water to prepare a 0.02% HA solution. The BC/CS membrane prepared in Section 2.3 was immersed in the 0.02% HA solution and stirred at 60 °C for 1 h. The membrane was then removed from the solution and washed with 400 mL of DI water at 60 °C for 1 h. The obtained membranes were named BS(CS/HA)_1_. For the second-round coating, the BS(CS/HA)_1_ membrane was immersed in a 0.1% CS solution and stirred at 60 °C for 1 h, followed by washing with DI water at 60 °C for 1 h. Subsequently, the membrane was immersed in the 0.1% Alg solution with stirring at 60 °C for 1 h and washed with 400 mL of DI water at 60 °C for 1 h. The coating cycle was repeated up to 10 times for lamination (BS(CS/HA)*_n_*, where n is the number of coating cycles). Eventually, the circular BC(CS/HA)_5_ and BC(CS/HA)_10_ membranes with a diameter of 8.5 cm were obtained and the thickness of the membranes is shown in Table 2.

### 2.5. Fourier-Transform Infrared (FT-IR) Spectroscopy

FT-IR measurements were performed on a JASCO FTIR-4200 instrument (Tokyo, Japan) using the KBr method to qualitatively analyze the compositions of the laminated membranes. The swollen membranes were freeze-dried before testing. A measurement range of 4000–400 cm^−1^, accumulation count of 32, and resolution of 2.0 cm^−1^ were used in all measurements.

### 2.6. Thermogravimetric Analysis (TGA)

Thermogravimetric analysis (TGA) was performed using an EXSTAR6000 instrument (Hitachi High-Tech Science Co., Ltd., Tokyo, Japan). The nitrogen flow rate was 200 mL min^−1^, the heating rate was 20 °C min^−1^, and the measurement temperature range was 22–600 °C. The moisture content of each sample was calculated from the TGA results. The reported values are the average of three measurements.

### 2.7. Scanning Electron Microscopy (SEM)

The sample surface was observed by field-emission scanning electron microscopy (FE-SEM; JSM6700, JEOL, Tokyo, Japan) after freezing the membranes in liquid nitrogen. The fiber diameter distribution and average fiber diameter were determined from 100 fibers from the SEM images using ImageJ.

### 2.8. Thickness

The sample thickness was measured in nine random locations (in the swollen state) using a digital micrometer (IP65, Mitutoyo, Kanagawa, Japan), and the average value was calculated and reported.

### 2.9. Tensile Properties

The tensile properties were tested using a material testing machine (STA-1150, A&D Co., Ltd., Tokyo, Japan). The swollen BC membrane was cut into strips with a width of 4.0 mm and length of 30.0 mm because it was difficult to shape it into a JIS dumbbell shape. The measurements were performed at a tensile speed of 10 mm/min, and the average tensile stress (N) and elongation at break (%) were obtained from 15 measurements. The tensile strength (Pa) was calculated using Equations (1) and (2).

(1) $$\begin{array}{c} \mathrm{Tensile}\;\mathrm{strength}\;(\mathrm{Pa})\;=\\ \mathrm{Average}\;\mathrm{tensile}\;\mathrm{strength}\;(N)/\mathrm{Average}\;\mathrm{cross}\text{-}\mathrm{sectional}\;\mathrm{area}\;(m^{2}), \end{array}$$

(2) $$\begin{array}{c} \mathrm{Average}\;\mathrm{cross}\text{-}\mathrm{sectional}\;\mathrm{area}\;(m^{2})\;=\\ \mathrm{Average}\;\mathrm{thickness}\;\mathrm{sample}\;\mathrm{in}\;\mathrm{swollen}\;\mathrm{state}\;(m)\;\times \;\mathrm{Sample}\;\mathrm{width}\;(m), \end{array}$$

The sample width was equal to 4 × 10^−3^ m.

### 2.10. Cytotoxicity Evaluation

#### 2.10.1. Cell Propagation for Cytotoxicity Test

BALB-3T3 clone A31 cells (mouse fibroblasts) were used as the cell model to analyze the cytotoxicity of the membrane by MTT method [37]. First, 4.7 g of EMEM and 0.75 g of NaHCO_3_ were dissolved in 440 mL of DI water. After that, 0.146 g of l-glutamic acid, 50 mL of FBS solution, and 10 mL of DI water were added. The obtained solution was sterilized and used as the new medium. Then, 1 mL of BALB-3T3 clone A31 cells was added to 10 mL of the sterilized medium and incubated at 37 °C for 24 h. Finally, the old medium was removed, replaced with fresh medium, and incubated for 72 h. The obtained solution was sterilized and referred to in this study as the medium. After incubation, the live cells were counted by a traditional cell counting method (counting cells by eye using a brightfield microscope). The dilution method yielded BALB-3T3 cells with a concentration of 1 × 10^4^ cells/well.

#### 2.10.2. Cytotoxicity Test

Prior to testing, small pieces of the sample with a diameter of 7 mm were sterilized by soaking in 70% ethanol for 3 h in triplicate. The sample was then soaked in PBS (pH 7.4) for 6 h in triplicate to remove the ethanol. Meanwhile, BALB-3T3 cells (1 × 10^4^ cells/well) were cultured as described in Section 2.10.1. Subsequently, 100 μL of BALB-3T3 cells were inoculated in 96-well plates and incubated for 1, 3, and 5 d under the same conditions. After incubation, the cell counts were determined using a Cell Counting Kit 8 (Dojindo, Kumamoto, Japan). The cell viability was calculated using Equation (3) according to the method of Oe et al. [37]:

Cell viability (%) = *Abs*_sample_/*Abs*_blank_ × 100, (3)

where *Abs*_sample_ and *Abs*_blank_ are the absorbances of samples with the laminated membrane and no membrane, respectively.

## 3. Results and Discussion

### 3.1. FT-IR Analysis

**Figure 3:**
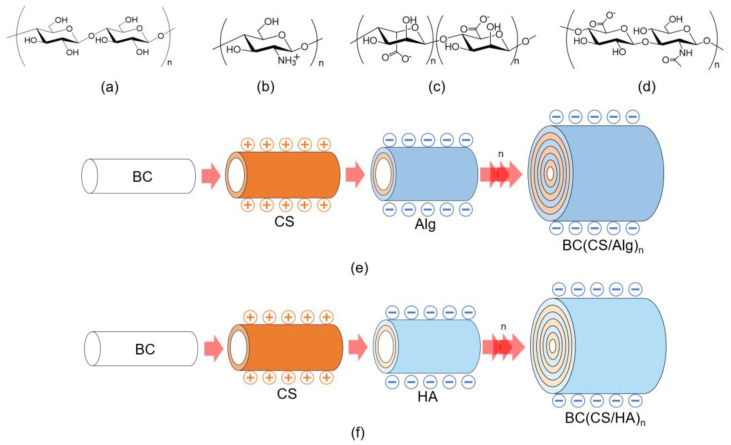
Chemical structures of (**a**) BC, (**b**) CS, (**c**) Alg, and (**d**) HA. Schematic illustrations of the (**e**) BC(CS/Alg) and (**f**) BC(CS/HA) membranes.

**Figure 4:**
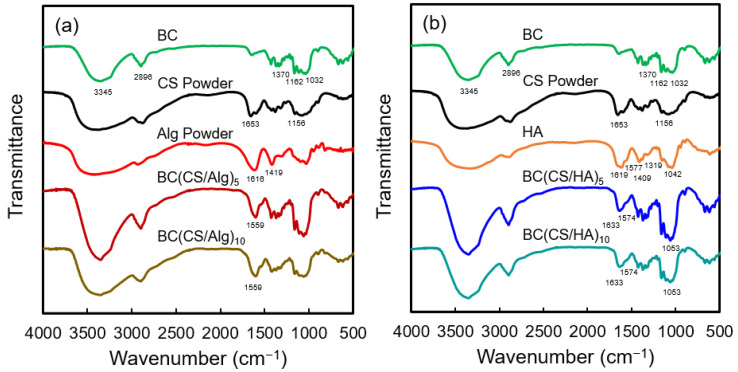
FT-IR spectra of (**a**) BC, CS powder, Alg powder, BC(CS/Alg)_5_, and BC(CS/Alg)_10_ membranes; (**b**) BC, CS powder, HA, BC(CS/HA)_5_, and BC(CS/HA)_10_ membranes.

The chemical structures of BC, CS, Alg, and HA and schematic illustrations of the BC(CS/Alg) and BC(CS/HA) membranes are shown in Figure 3a–f, respectively. The FT-IR spectra of the BC, BC(CS/Alg)_5_, BC(CS/Alg)_10_, BC(CS/HA)_5_, and BC(CS/HA)_10_ membranes are shown in Figure 4. A typical spectrum was observed for BC with peaks at 3345 cm^−1^ (–OH), 2896 cm^−1^, 1370 cm^−1^ (–CH), 1032 cm^−1^ (β-1,4 glycosidic bond, C–O–C), and 1162 cm^−1^ (C–O–C stretching) [38]. CS exhibited peaks at 1156 cm^−1^ (C–N stretching) and 1653 cm^−1^ (amide I) [2]. Alg powder peaks were observed at 1616 and 1419 cm^−1^, corresponding to the carboxylic group (–COO^−^) [23]. For unmodified HA, peaks are typically present at 1666 and 1659 cm^−1^, corresponding to the C–N stretching of amide I and II, respectively. Moreover, peaks are typically observed at 1619, 1417, and 1322 cm^−1^, which are attributed to the stretching of asymmetric and symmetric bands of the carboxylate groups and the stretching of C–N [39]. In this study, pure HA exhibited peaks at 1619, 1409, and 1319 cm^−1^. Furthermore, a shoulder was observed at 1577 cm^−1^, which was attributed to amide II, and another characteristic peak of HA was observed at ~1042 cm^−1^ [38]. The characteristic peaks of BC were observed in all the laminated membranes.

For the BC(CS/Alg)_5_ and BC(CS/Alg)_10_ membranes (Figure 4a), a peak was observed at 1599 cm^−1^, possibly representing the electrostatic interaction between the –COO^−^ group of Alg and –NH_3_^+^ group of CS. In addition, the peak at 1736 cm^−1^ (non-ionized COOH) was not present, possibly because the non-ionized COOH group changed to –COO^−^ in the presence of the –NH_3_^+^ group of CS [24]. The two membranes did not exhibit any difference in peaks. The FT-IR spectra confirmed that coating CS and Alg on BC using the LBL method was successful. A schematic of the BC(CS/Alg) membrane structure and interactions is shown in Figure 3e.

For the BC(CS/HA)_5_ and BC(CS/HA)_10_ membranes (Figure 4b), the peak at 1633 cm^−1^ represents the amide I of either CS or HA. A shoulder at 1574 cm^−1^, corresponding to the amide II of HA, and a characteristic peak of HA at 1053 cm^−1^ were observed in both membranes [38]. The shifted peaks of amide I and II from pure CS powder and pure HA imply an interaction between CS and HA. The two membranes did not exhibit any difference in peaks. According to the FT-IR spectra, no relevant peak differences can be observed between HA and non-ionized HA [40]. Therefore, the FT-IR spectra only confirmed the functional groups of BC, CS, and HA in the BC(CS/HA) membrane. The electrostatic interactions between CS (cation) and HA (anion) have been studied and reported previously [40]. HA is usually considered an anionic polysaccharide. HA consists of repeating disaccharide subunits of anionic d-glucuronic acid and weakly cationic *N*-acetyl-d-glucosamine, which give HA a very low isoelectric point of approximately 2.5 [41]. The electrostatic interactions between CS (cation) and HA (anion) are believed to be the main reason for the successful preparation of the BC(CS/HA) membrane, as shown in Figure 3f.

### 3.2. Thermal Analysis

**Figure 5:**
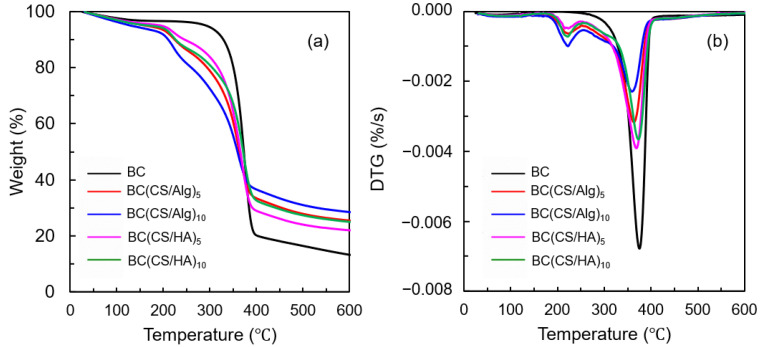
(**a**) TGA curves and (**b**) differential thermogravimetric curves of BC, BC(CS/Alg)_5_, BC(CS/Alg)_10_, BC(CS/HA)_5_, and BC(CS/HA)_10_ membranes.

The thermal stabilities of the BC, BC(CS/Alg)_5_, BC(CS/Alg)_10_, BC(CS/HA)_5_, and BC(CS/HA)_10_ membranes were reflected by their mass-loss profiles and degradation rates obtained from TGA. The TGA and differential thermogravimetric curves for each membrane are shown in Figure 5a,b, respectively. From the initial stage until 110 °C, broad weight loss peaks were observed for all samples, representing water evaporation. The moisture content of each sample is listed in Table 2. The pristine BC membrane exhibited a one-step decomposition at 374 °C, corresponding to the dehydration and decomposition of cellulose molecules [24]. Weight loss peaks were observed at 224 and 222 °C for the BC(CS/Alg)_5_ and BC(CS/Alg)_10_ membranes, respectively, belonging to the decomposition of sodium Alg [42], while the weight loss peaks of the BC(CS/HA)_5_ and BC(CS/HA)_10_ membranes appeared at 224 and 222 °C, respectively, owning to the polysaccharide degradation of HA at 225 °C [38]. The decomposition peak of CS at 305 °C was not present in all the laminated membranes [24]. Meanwhile, the decomposition peak of BC, which typically occurs at around 374 °C, shifted to a lower temperature for all laminated membranes, implying that the interaction between CS and BC occurred successfully.

### 3.3. Physical Appearance and Morphology of the Membrane

**Figure 6:**
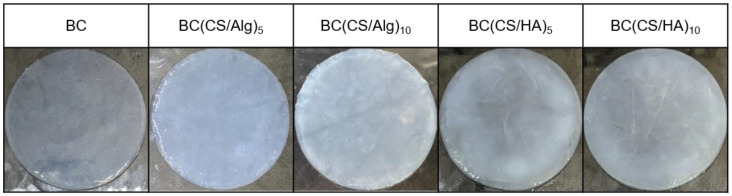
The physical appearance of the membrane surface of BC, BC(CS/Alg)_5_, BC(CS/Alg)_10_, BC(CS/HA)_5_, and BC(CS/HA)_10_ membranes.

**Figure 7:**
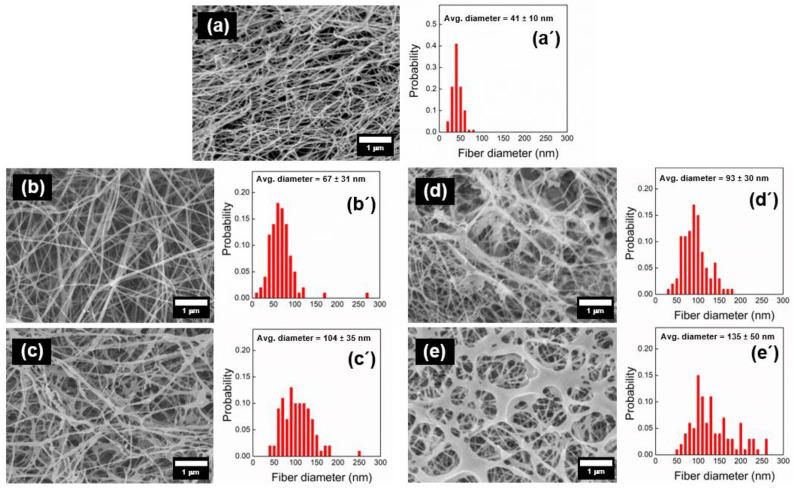
(**a**–**e**) SEM images of the membrane surface and (**a**’–**e**’) fiber diameter distribution of (**a**,**a**’) BC, (**b**,**b**’) BC(CS/Alg)_5_, (**c**,**c**’) BC(CS/Alg)_10_, (**d**,**d**’) BC(CS/HA)_5_, and (**e**,**e**’) BC(CS/HA)_10_ membranes.

The physical appearance of each membrane is shown in Figure 6. The SEM images of the membrane surfaces and the fiber diameter distributions are shown in Figure 7. The fiber diameters were determined from the SEM images using ImageJ. The BC membrane (Figure 7a) comprised an interwoven mesh with an average fiber diameter of 41 nm, which is typical for BC materials [38]. The BC(CS/Alg)_5_ membrane contained relatively thin fibers with an average diameter of 67 ± 31 nm, whereas the fibers in the BC(CS/Alg)_10_ membrane had an average diameter of 104 ± 36 nm. As for the BC(CS/Alg) membranes, the average fiber diameter was smaller for the BC(CS/HA)_5_ membrane (93 ± 30 nm) than that for the BC(CS/HA)_10_ membrane (135 ± 50 nm). These results agree with those of Huang et al., who studied CS/tannic acid-deposited cellulose nanofibrous mats and found that the average fiber diameter increased with the number of layers [2].

### 3.4. Thickness in the Swollen State, Moisture Content and Tensile Properties

**Figure 8:**
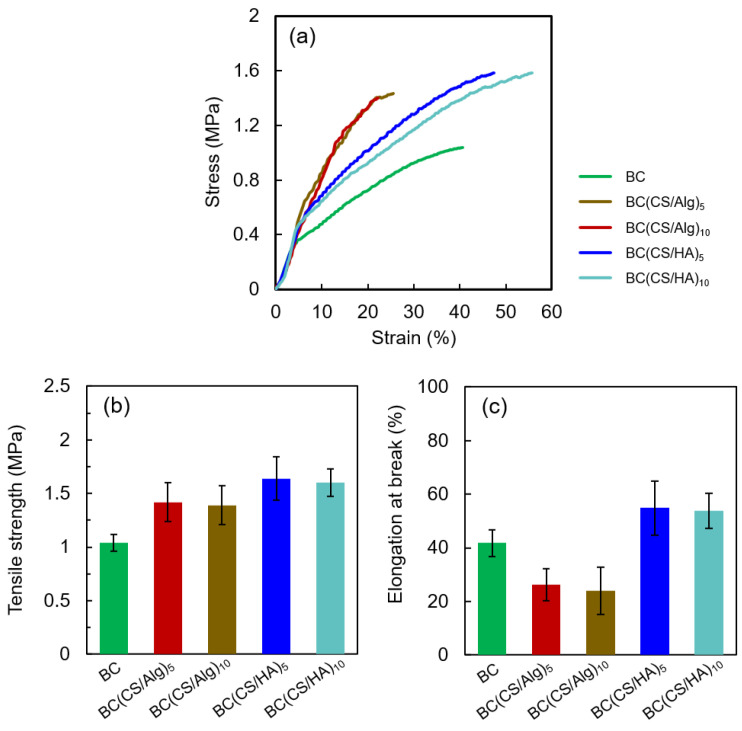
(**a**) Stress–Strain curves, (**b**) tensile strength, and (**c**) elongation at break of BC, BC(CS/Alg)_5_, BC(CS/Alg)_10_, BC(CS/HA)_5_, and BC(CS/HA)_10_ membranes.

The thicknesses of the membranes in the swollen and dried states and the moisture contents of the dried membranes are listed in Table 2, and their tensile properties are shown in Figure 8. Herein, the laminated membranes in the swollen state were only used for the tensile strength measurements. As shown in Table 2, since the number of layers increased, the thickness of both dried BC(CS/Alg) membranes and BC(CS/HA) membranes tended to increase, which aligns with previous reports [43]. In addition, the laminated membranes’ moisture contents were higher than that of the BC membrane. The tensile strength and elongation at break results can be divided into three ranges based on the raw materials. The tensile strength of the BC membrane was 1.04 MPa, and the elongation at break was approximately 42%. Laminating with CS and Alg increased the tensile strength (from 1.04 to 1.39–1.42 MPa) but decreased the elongation at break (from 42 to 25%), which contrasts with the results of previous works. Cao et al. reported that the elongation at break of bilayer sodium alginate (SA)/CS membranes (28.74 ± 1.47%) was higher than that of single-layer CS and SA membranes (22.38 ± 0.52% and 8.25 ± 1.24%, respectively), which agreed with the results of previous work [44,45]. However, the bilayer SA/CS membranes in these works were fabricated with glycerol as a plasticizer [44,45]; by contrast, in this study, the BC(CS/Alg) membranes were fabricated without plasticizer, so the decrease in elongation at break (as compared to the bare BC membrane) is understandable. Interestingly, laminating with HA and CS increased both the tensile strength (from 1.04 to 1.60–1.64 MPa) and elongation at break (from 42 to 54%). To our knowledge, there is little research on the tensile properties of bilayer and multilayer CS/HA membranes. Yao and Wu (2010) found that the tensile strength and extensibility of the CS/HA-blended membranes decreased with increasing HA content when the blending ratio was under 1:0.05. By contrast, the tensile strength and extensibility increased with increasing HA content to above that of a pure CS membrane when the CS/HA-blending ratio was above 1:0.1 [46]. The enhanced elongation of the BC(CS/HA) membranes implies that CS/HA ratio in the membrane is optimal for improving the tensile properties. The increase in tensile strength in the BC(CS/Alg) and BC(CS/HA) membranes is believed to be due to the interactions between CS and Alg and between CS and HA, respectively [22,40]

### 3.5. Cytotoxicity Analysis

**Figure 9:**
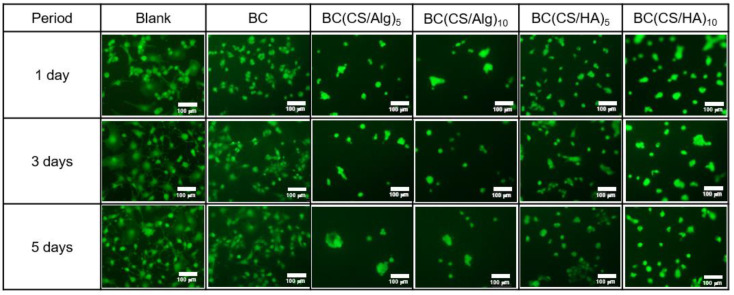
Representative fluorescence micrographs of live strained blank, BC, BC(CS/Alg)_5_, BC(CS/Alg)_10_, BC(CS/HA)_5_, and BC(CS/HA)_10_ membranes at 1, 3, and 5 d.

**Figure 10:**
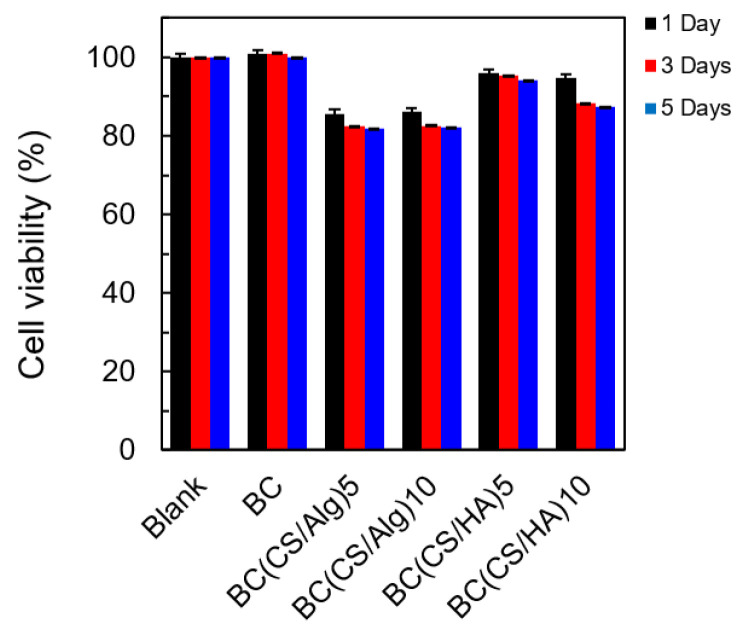
Cell viability percentage of blank, BC, BC(CS/Alg)_5_, BC(CS/Alg)_10_, BC(CS/HA)_5_, and BC(CS/HA)_10_ membranes at 1, 3, and 5 d.

The cytotoxicity was analyzed using BALB-3T3 clone A31 cells (mouse fibroblasts, 1 × 10^4^ cells/well). The BALB-3T3 clone A31 cells were cultured for 1 week. After that, 100 μL of BALB-3T3 cells were inoculated in 96-well plates and incubated for 1, 3, and 5 d. Representative fluorescence micrographs of live strained membranes and the viability percentage of each membrane at 1, 3, and 5 d are shown in Figure 9 and Figure 10, respectively. A representative fluorescence micrograph of the blank culture (without any membrane) exhibited the same number of live cells at 1, 3, and 5 d (100% cell viability). For the BC membrane (control), the cell viability was around 100%, similar to the blank culture. By contrast, the cell viabilities of the BC(CS/Alg)_5_ and BC(CS/Alg)_10_ membranes were both approximately 82% after 5 d, whereas the BC(CS/HA)_5_ and BC(CS/HA)_10_ membranes yielded cell viabilities of 94 and 87% under the same conditions. The higher biocompatibility of the BC(CS/HA) membranes was ascribed to the ability of HA to reduce the injury to cells caused by CS and to improve the biocompatibility of the membrane [46]. Our finding is similar to that of Tamer et al., who reported that the cytotoxicity of CS/HA membranes and CS/HA/glutathione membranes showed the cell viability (NIH3T3, mouse fibroblast cells with the initial concentration of 4 × 10^3^ cells/well) at 2 d of 93.1 and 94.4%, respectively. The percentages of viable cells were calculated comparing with the control culture without the membranes [47]. In summary, the BC(CS/HA)_5_ membrane exhibited the highest biocompatibility of the laminated membranes, with a cell viability of 94% at 5 d, which is higher than the 92%, indicating that this membrane is safe for wound dressing [20]. This result also showed the high possibility to add some antibiotic substances to either CS or HA layers to improve the wound healing property of this BC(CS/HA)_5_ membrane.

## 4. Conclusions

BC(CS/HA) and BC(CS/Alg) membranes were successfully prepared via the LBL method and characterized. The decomposition temperatures from the TGA and FT-IR spectra confirmed the electrostatic interactions between CS and Alg in the BC(CS/Alg) membrane and the interactions between CS and HA in the BC(CS/HA) membrane. SEM images revealed that the fiber diameters of the BC(CS/Alg) and BC(CS/HA) membranes were larger than those of the BC membrane. In addition, the BC(CS/Alg) membrane exhibited higher tensile strength but lower elongation at break as compared to the BC membrane, whereas the BC(CS/HA) membrane had improved tensile strength and elongation at break compared to the BC membrane. The BC(CS/HA)_5_ membrane exhibited the highest biocompatibility with a cell viability of 94% at 5 d, which is higher than 92%, indicating its potential for use as a wound dressing material. However, in vivo evaluation is suggested to further study in the future.

## Figures and Tables

**Table 1:** Details of each sample.

Sample Name	Coating Cycles
BC	-
BC(CS/Alg)_5_	5
BC(CS/Alg)_10_	10
BC(CS/HA)_5_	5
BC(CS/HA)_10_	10

**Table 2:** Thickness of BC and laminated membranes in the swollen states and moisture content of the dried BC and laminated membranes.

Sample Name	Thickness (mm) in the Swollen State	Thickness (mm) of Dried Membrane	Moisture Content (%) of Dried Membrane
BC	0.49 ± 0.08	0.007 ± 0.004	3.20 ± 0.07
BC(CS/Alg)_5_	0.49 ± 0.06	0.042 ± 0.013	4.36 ± 0.13
BC(CS/Alg)_10_	0.34 ± 0.04	0.062 ± 0.022	4.73 ± 0.23
BC(CS/HA)_5_	0.30 ± 0.06	0.015 ± 0.005	3.99 ± 0.06
BC(CS/HA)_10_	0.32 ± 0.06	0.027 ± 0.015	4.25 ± 0.10

## Data Availability

Data will be made available on request.

## References

[1] Ding F., Deng H., Du Y., Shi X., Wang Q. (2014). Emerging chitin and chitosan nanofibrous materials for biomedical applications. Nanoscale.

[2] Huang J., Cheng Y., Wu Y., Shi X., Du Y., Deng H. (2019). Chitosan/tannic acid bilayers layer-by-layer deposited cellulose nanofibrous mats for antibacterial application. Int. J. Biol. Macromol..

[3] Husteden C., Doberenz F., Goergen N., Pinnapireddy S.R., Janich C., Langner A., Syrowatka F., Repanas A., Erdmann F., Jedelská J. (2020). Contact-triggered lipofection from multilayer films designed as surfaces for in situ transfection strategies in tissue engineering. ACS Appl. Mater. Interfaces.

[4] Panda P.K., Yang J.-M., Chang Y.-H. (2021). Preparation and characterization of ferulic acid-modified water soluble chitosan and poly (gamma-glutamic acid) polyelectrolyte films through layer-by-layer assembly towards protein adsorption. Int. J. Biol. Macromol..

[5] Gan M., Guo C., Liao W., Liu X., Wang Q. (2023). Development and characterization of chitosan/bacterial cellulose/pullulan bilayer film with sustained release curcumin. Int. J. Biol. Macromol..

[6] Ishihara M., Nakanishi K., Ono K., Sato M., Kikuchi M., Saito Y., Yura H., Matsui T., Hattori H., Uenoyama M. (2002). Photocrosslinkable chitosan as a dressing for wound occlusion and accelerator in healing process. Biomaterials.

[7] Ahmed S., Ikram S. (2016). Chitosan based scaffolds and their applications in wound healing. Achiev. Life Sci..

[8] Singh R., Shitiz K., Singh A. (2017). Chitin and chitosan: Biopolymers for wound management. Int. Wound J..

[9] Wang J., Tavakoli J., Tang Y. (2019). Bacterial cellulose production, properties and applications with different culture methods—A review. Carbohydr. Polym..

[10] Fontana J.D., De Souza A.M., Fontana C.K., Torriani I.L., Moreschi J.C., Gallotti B.J., De Souza S.J., Narcisco G.P., Bichara J.A., Farah L.F.X. (1990). Acetobacter cellulose pellicle as a temporary skin substitute. Appl. Biochem. Biotechnol..

[11] Czaja W., Krystynowicz A., Bielecki S., Brown R.M. (2006). Microbial cellulose—The natural power to heal wounds. Biomaterials.

[12] Kwak M.H., Kim J.E., Go J., Koh E.K., Song S.H., Son H.J., Kim H.S., Yun Y.H., Jung Y.J., Hwang D.Y. (2015). Bacterial cellulose membrane produced by Acetobacter sp. A10 for burn wound dressing applications. Carbohydr. Polym..

[13] Sun J., Tan H. (2013). Alginate-based biomaterials for regenerative medicine applications. Materials..

[14] Silva J., Vanat P., Marques-da-Silva D., Rodrigues J.R., Lagoa R. (2020). Metal alginates for polyphenol delivery systems: Studies on crosslinking ions and easy-to-use patches for release of protective flavonoids in skin. Bioact. Mater..

[15] Kamoun E.A., Kenawy E.R.S., Tamer T.M., El-Meligy M.A., Eldin M.S.M. (2015). Poly(vinyl alcohol)-alginate physically crosslinked hydrogel membranes for wound dressing applications: Characterization and bio-evaluation. Arab. J. Chem..

[16] Ozaki C.K., Hamdan A.D., Barshes N.R., Wyers M., Hevelone N.D., Belkin M., Nguyen L.L. (2015). Prospective, randomized, multi-institutional clinical trial of a silver alginate dressing to reduce lower extremity vascular surgery wound complications. J. Vasc. Surg..

[17] Dowling M.B., Chaturvedi A., MacIntire I.C., Javvaji V., Gustin J., Raghavan S.R., Scalea T.M., Narayan M. (2016). Determination of efficacy of a novel alginate dressing in a lethal arterial injury model in swine. Injury.

[18] Kurczewska J., Pecyna P., Ratajczak M., Gajęcka M., Schroeder G. (2017). Halloysite nanotubes as carriers of vancomycin in alginate-based wound dressing. Saudi Pharm. J..

[19] Archana D., Dutta J., Dutta P.K. (2013). Evaluation of chitosan nano dressing for wound healing: Characterization, in vitro and in vivo studies. Int. J. Biol. Macromol..

[20] Xue H., Hu L., Xiong Y., Zhu X., Wei C., Cao F., Zhou W., Sun Y., Endo Y., Liu M. (2019). Quaternized chitosan-Matrigel-polyacrylamide hydrogels as wound dressing for wound repair and regeneration. Carbohydr. Polym..

[21] Hu B., Guo Y., Li H., Liu X., Fu Y., Ding F. (2021). Recent advances in chitosan-based layer-by-layer biomaterials and their biomedical applications. Carbohydr. Polym..

[22] Li K., Zhu J., Guan G., Wu H. (2019). Preparation of chitosan-sodium alginate films through layer-by-layer assembly and ferulic acid crosslinking: Film properties, characterization, and formation mechanism. Int. J. Biol. Macromol..

[23] Ibrahim A., Khalil I.A., Mahmoud M.Y., Bakr A.F., Ghoniem M.G., Al-Farraj E.S., El-Sherbiny I.M. (2023). Layer-by-layer development of chitosan/alginate-based platelet-mimicking nanocapsules for augmenting doxorubicin cytotoxicity against breast cancer. Int. J. Biol. Macromol..

[24] Kotatha D., Morishima K., Uchida S., Ogino M., Ishikawa M., Furuike T., Tamura H. (2018). Preparation and characterization of gel electrolyte with bacterial cellulose coated with alternating layers of chitosan and alginate for electric double-layer capacitors. Res. Chem. Intermed..

[25] Gomes A.P., Mano J.F., Queiroz J.A., Gouveia I.C. (2012). Layer-by-layer deposition of antibacterial polyelectrolytes on cotton fibres. J. Polym. Environ..

[26] Gomes A.P., Mano J.F., Queiroz J.A., Gouveia I.C. (2015). Incorporation of antimicrobial peptides on functionalized cotton gauzes for medical applications. Carbohydr. Polym..

[27] Huang R., Li W., Lv X., Lei Z., Bian Y., Deng H., Wang H., Li J., Li X. (2015). Biomimetic LBL structured nanofibrous matrices assembled by chitosan/collagen for promoting wound healing. Biomaterials.

[28] Wu G., Ma X., Fan L., Gao Y., Deng H., Wang Y. (2020). Accelerating dermal wound healing and mitigating excessive scar formation using LBL modified nanofibrous mats. Mater. Des..

[29] Mandapalli P.K., Labala S., Bojja J., Venuganti V.V.K. (2016). Effect of pirfenidone delivered using layer-by-layer thin film on excisional wound healing. Eur. J. Pharm. Sci..

[30] Prasathkumar M., Sadhasivam S. (2021). Chitosan/hyaluronic acid/alginate and an assorted polymers loaded with honey, plant, and marine compounds for progressive wound healing—Know-how. Int. J. Biol. Macromol..

[31] Zhai P., Peng X., Li B., Liu Y., Sun H., Li X. (2020). The application of hyaluronic acid in bone regeneration. Int. J. Biol. Macromol..

[32] Luo Z., Dai Y., Gao H. (2019). Development and application of hyaluronic acid in tumor targeting drug delivery. Acta Pharm. Sin. B.

[33] Al-Khateeb R., Olszewska-Czyz I. (2020). Biological molecules in dental applications: Hyaluronic acid as a companion biomaterial for diverse dental applications. Heliyon.

[34] Lafuente-Merchan M., Ruiz-Alonso S., Espona-Noguera A., Galvez-Martin P., López-Ruiz E., Marchal J.A., López-Donaire M.L., Zabala A., Ciriza J., Saenz-del-Burgo L. (2021). Development, characterization and sterilisation of nanocellulose-alginate-(hyaluronic acid)- bioinks and 3D bioprinted scaffolds for tissue engineering. Mater. Sci. Eng. C.

[35] Li Y., Jiang H., Zheng W., Gong N., Chen L., Jiang X., Yang G. (2015). Bacterial cellulose–hyaluronan nanocomposite biomaterials as wound dressings for severe skin injury repair. J. Mater. Chem. B.

[36] del Hoyo-Gallego S., Pérez-Álvarez L., Gómez-Galván F., Lizundia E., Kuritka I., Sedlarik V., Laza J.M., Vila-Vilela J.L. (2016). Construction of antibacterial poly(ethyleneterephthalate) films via layer by layer assembly of chitosan and hyaluronic acid. Carbohydr. Polym..

[37] Oe T., Dechojarassri D., Kakinoki S., Kawasaki H., Furuike T., Tamura H. (2023). Microwave-assisted incorporation of AgNP into chitosan–alginate hydrogels for antimicrobial applications. J. Funct. Biomater..

[38] Wang X., Tang J., Huang J., Hui M. (2020). Production and characterization of bacterial cellulose membranes with hyaluronic acid and silk sericin. Colloids Surf. B Biointerfaces.

[39] Maiz-Fernández S., Pérez-Álvarez L., Silván U., Vilas-Vilela J.L., Lanceros-Méndez S. (2022). Dynamic and self-healable chitosan/hyaluronic acid-based in situ-forming hydrogels. Gels.

[40] Yang P.-F., Lee C.-K. (2007). Hyaluronic acid interaction with chitosan-conjugated magnetite particles and its purification. Biochem. Eng. J..

[41] Gatej I., Popa M., Rinaudo M. (2005). Role of the pH on hyaluronan behavior in aqueous solution. Biomacromolecules.

[42] Liu S., Li Y., Li L. (2017). Enhanced stability and mechanical strength of sodium alginate composite films. Carbohydr. Polym..

[43] Neto A.I., Cibrão A.C., Correia C.R., Carvalho R.R., Luz G.M., Ferrer G.G., Botelho G., Picart C., Alves N.M., Mano J.F. (2014). Nanostructured polymeric coatings based on chitosan and dopamine-modified hyaluronic acid for biomedical applications. Small.

[44] Cao S., Wang S., Wang W., Lin G., Niu B., Guo R., Yan H., Wang H. (2023). Sodium alginate/chitosan-based intelligent bilayer film with antimicrobial activity for pork preservation and freshness monitoring. Food Control.

[45] Wang H., Gong X., Miao Y., Guo X., Liu C., Fan Y.-Y., Zhang J., Niu B., Li W. (2019). Preparation and characterization of multilayer films composed of chitosan, sodium alginate and carboxymethyl chitosan-ZnO nanoparticles. Food Chem..

[46] Yao Z.-A., Wu H.-G. Characterization of chitosan-hyaluronic acid blended membranes and their biocompatibility with keratocytes. Proceedings of the 3rd International Conference on Biomedical Engineering and Informatics.

[47] Tamer T.M., Hassan M.A., Valachová K., Omer A.M., El-Shafeey M.E.A., Mohy Eldin M.S., Šoltés L. (2020). Enhancement of wound healing by chitosan/hyaluronan polyelectrolyte membrane loaded with glutathione: In Vitro and in vivo evaluations. J. Biotech..

